# The role of personality in body image dissatisfaction and disordered eating: discrepancies between men and women

**DOI:** 10.1186/s40337-017-0177-8

**Published:** 2017-10-18

**Authors:** L. P. MacNeill, L. A. Best, L. L. Davis

**Affiliations:** Deparment of Psychology, University of New Brunswick, Saint John, 100 Tucker Park Road, P.O. Box 5050, Saint John, NB E2L 4L5 Canada

**Keywords:** Gender differences, Big five personality factors, Disordered eating, Body image dissatisfaction

## Abstract

**Background:**

Body image and disordered eating research has focused mostly on the female experience. The present study examined gender differences in the relationship between personality, disordered eating, and body image dissatisfaction.

**Methods:**

Participants were 238 female and 85 male undergraduates (*M*_age_ = 20.52 years, *SD* = 4.22) at a Canadian university. Materials included a battery of self-report questionnaires pertaining to personality, body image, and disordered eating.

**Results:**

As expected, females reported more body dissatisfaction and disordered eating than males. Personality factors were found to be significantly related to the experience of body dissatisfaction in both genders. Further, several personality traits significantly contributed to the prediction of male (high Neuroticism, low Conscientiousness) and female (high Neuroticism) body dissatisfaction beyond the influence of body mass index (BMI). Interestingly, and contrary to findings with female participants, personality traits were not significantly related to disordered eating scores in men. Among women, disordered eating scores were significantly predicted by high Neuroticism and Extraversion, and low Conscientiousness.

**Conclusions:**

Although the relationship between disordered eating, body image dissatisfaction, and personality is well-documented in females, this relation may differ for males. The focus on male body image has been increasing in Western society; exploring how males view their bodies may be beneficial to researchers and clinicians alike.

## Plain English summary

Body image concerns do not discriminate; body dissatisfaction and its consequences are relevant to men and women of all ages. Traditionally, disordered eating and body image research has been conducted with populations of young females; therefore, more studies that concentrate on both male and female experiences of body dissatisfaction and disordered eating attitudes and behaviours are needed. Our study examined associations between body dissatisfaction, disordered eating, and personality in both genders. Several of our findings were consistent with those reported in previous studies: women reported higher levels of body dissatisfaction and disordered eating behaviours compared to men; and, personality traits were related to the experience of body dissatisfaction in both genders. Interestingly, the association between disordered eating and personality traits differed for men and women such that personality was associated with disordered eating in women, but not in men. These findings suggest that male and female experiences of body dissatisfaction and disordered eating may differ in important, clinically relevant ways. A further exploration of these differences could be beneficial in informing the prevention and treatment of body image concerns and disordered eating attitudes and behaviours in both genders.

## Background

In the past, body image research had primarily investigated body dissatisfaction and disordered eating in women; however, the focus on the study of men has been increasing. Perceptions of the ideal female body may be altered by frequent media exposure to thin female bodies [1–3], which may lead women to experience more body dissatisfaction and body focus than men [2–8]. Moreover, body dissatisfaction in women has been consistently associated with disordered eating behaviours and the development of eating disorders [9–12].

For women in particular, disordered eating behaviours have become so common in Western society that dieting is widely considered *normal* [13]. Given their pervasiveness in Western society, researchers have investigated various psychological correlates of body dissatisfaction and disordered eating in an attempt to elucidate their etiology. Gender differences have received less attention; however, gender differences among these relationships may indicate that male and female experiences of body dissatisfaction and disordered eating differ in important, clinically relevant ways. Previous literature suggests that significant variance in the development, onset, and maintenance of eating disorders can be attributed to personality factors [14, 15]. Therefore, the current study aimed to elucidate potential gender differences in the relationship between body dissatisfaction, disordered eating, and the five-factor model (FFM) of personality in a sample of Canadian undergraduate students. One purpose of this study was to determine whether specific predictors of body dissatisfaction and disordered eating were similar for males and females. Although there is a growing body of research on body dissatisfaction and disordered eating attitudes among males, very little research has included examinations of how personality affects these outcomes. To our knowledge, this study is one of the few that examines the influence of personality after controlling for body mass index (BMI) in a sample of male participants.

### Gender differences in body dissatisfaction and disordered eating

The importance of physical appearance is more emphasized for women, and appearance norms are especially pervasive in Western society where they are conveyed through a variety of social agents, most notably the mass media [16–18]. As a result, women often experience greater body focus and body dissatisfaction than men [3, 5, 8, 19]. Women consistently choose ideal figures that are smaller than their perceived current size [2, 3, 6–8], whereas males choose ideal body sizes that are similar to, or larger than, their perceived current size [8, 20, 21]. In a study by Grieve, Newton, Kelley, Miller, and Kerr [22], men chose an ideal male body that was more muscular than their current body and thought that women would prefer a more muscular male body than was actually preferred by females.

Further, it has been found that a participant’s BMI category is related to gender differences in body dissatisfaction. The relationship between BMI and body dissatisfaction is linear for women, meaning women become more dissatisfied with their bodies as their BMI increases [23]. This relationship for men, however, is more complex; men with either a low BMI or a high BMI are typically more dissatisfied with their bodies than men who fall in a middle range of the BMI [2, 23]. Very thin women approach the thin societal ideal for women and would therefore be more satisfied with their appearance, whereas very thin men, would deviate farther from the muscular male ideal of Western society and would therefore show more dissatisfaction.

It is believed that gender differences in disordered eating are also influenced by the idealization of thinness for women in Western Society [24–26]. Anorexia Nervosa and Bulimia Nervosa are characterized by abnormal eating behaviours, weight regulation, and distorted attitudes and perceptions about body weight and shape, and both disorders are more common in women [10, 27, 28]. In a recent review of studies assessing eating disorder prevalence [29], results indicated that in two-stage design studies, point prevalence was most frequently described with overall eating disorder rates for females ranging from 0.5% [30] to 5.3% [31], and those for males ranging from 0.62% [32] to 0.64% [31]. Data reveals that females are more likely than males to respond to their weight concerns by engaging in dieting behaviours, binge eating, and purging behaviours [11, 33, 34]. In contrast, men are more likely to use other activities such as exercise for weight control and these are not captured or discussed in men’s assessments [11, 33, 35–37].

In recent decades, the media focus on male bodies has been increasing, which may lead to a shift in male body perceptions (i.e., the muscular ideal) [7, 22, 38, 39]. With the increasing pressure on men to attain certain societal ideals, disordered eating in men could increase and this muscular ideal could minimize the gap in body image concerns and disordered eating among men and women [7].

### Personality correlates of body dissatisfaction and disordered eating

Many researchers agree that five basic dimensions explain individual differences in personality [40, 41]. This model includes the personality traits Neuroticism, Extraversion, Openness to experience, Agreeableness, and Conscientiousness [42, 43]. Using the FFM, research with women has demonstrated that body image dissatisfaction and disordered eating are consistently related to higher levels of neuroticism and lower levels of extraversion [44–49].

For example, Dalley et al. [44] investigated the relationship between body image dissatisfaction and neuroticism, and found that neuroticism is a salient factor in the development of body dissatisfaction in women. Neuroticism has also been shown to intensify the negative effects of other variables, such as BMI [50, 51]. Likewise, Swami et al. [52] used the FFM to investigate the relationship between personality and body dissatisfaction and found that neuroticism was positively related to level of body dissatisfaction, while extraversion was negatively related to level of body dissatisfaction.

The FFM has also been applied to disordered eating attitudes and behaviours in women [45, 46]. For instance, Miller et al. [46] examined the relationship between neuroticism, extraversion, and eating attitudes in women and found that neuroticism was positively related to disordered eating, and extraversion was negatively related to disordered eating. Similarly, MacLaren and Best [45] investigated the relationship between personality and eating attitudes in female university students and found that disordered eating attitudes were related to high neuroticism and low extraversion. Although researchers have explored these personality correlates of eating attitudes and behaviours in women, to date, little research has been done examining these correlates in men who have similar levels of body dissatisfaction and disordered eating attitudes.

### The current study

The current paper focuses on the examination of potential gender differences in the relationship between body dissatisfaction, eating attitudes, and the FFM of personality. Males have not been the typical focus of body image and disordered eating research and, to date, no published studies have investigated these factors in both genders. Although clinical samples are optimal, given the relatively low prevalence of ED in males and females, obtaining a sample with sufficient power would be very difficult. We feel this research is an important first step that can elucidate important correlates and inform future clinical research. Further, even in non-clinical populations, negative attitudes about body image and eating can lead to adverse mental and physical health outcomes and prevalence rates for disordered eating and body dissatisfaction are high in university samples.

Hypotheses:

1. Women would report greater levels of body dissatisfaction and disordered eating than men.
2. Personality scores would predict levels of disordered eating in women. Given the lack of literature, no specific prediction was made for men.
3. Personality scores would predict levels of body dissatisfaction in women. Given the lack of literature, no specific prediction was made for men.

Although our results are preliminary, the current report provides a broader and more dynamic picture of gender differences in body image and eating behaviours.

## Methods

### Participants

Participants (*N* = 323) were undergraduate students at a small Atlantic Canadian university enrolled in introductory-level psychology courses. Participants were recruited over a two-year time period through poster advertisements on campus, and were offered course credit for their participation. Participants took part in the study in small groups ranging in size from 2 to 20 individuals, depending on participant availability. During the data collection session, participants completed a battery of self-report questionnaires in random order, where a demographics sheet always appeared first. Written informed consent was given by all participants and all study procedures were approved by the University’s Research Ethics Board.

### Measures

#### Disordered eating

The Eating Attitudes Test-26 (EAT-26) [53] contains 26 self-report items and measures atypical behaviours and attitudes about eating (e.g., “Find myself preoccupied with food”; “Avoid eating when I am hungry”). These thoughts, feelings, and behaviours are scored on a 6-point Likert scale ranging from 1 (*always*) to 6 (*never*). Scores were reverse coded such that higher EAT-26 scores are indicative of higher levels of disordered eating. The EAT-26 has demonstrated good reliability and validity in males and females and is an adequate screening tool in non-clinical settings [53, 54]. In the current study, the internal reliability ranged from α = .88 for females and α = .83 for males.

#### Body image dissatisfaction

The Body Image Ideals Questionnaire (BIQ) [55] is a 22-item self-report questionnaire that measures the self-perceived discrepancy between internalized ideals and current body condition in regards to 11 physical characteristics: height; skin complexion; hair texture and thickness; facial features; muscle tone and definition; body proportions; weight; chest (or breast) size; physical strength; physical coordination; and, overall appearance (e.g., “My ideal muscle tone and definition is:”). In Part A, each physical characteristic is rated on a 4 point Likert scale ranging from 0 (*exactly as I am*) to 3 (*very unlike me*). In Part B, each physical characteristic is again rated in terms of importance (e.g., “How important to you is your ideal physical coordination?”). Each item is rated on a 4 point Likert scale ranging from 0 (*not important*) to 3 (*very important*). The mean of the item-by-item cross-products of discrepancy by importance ratings is then calculated for each participant. The BIQ has demonstrated good reliability and validity for women and men respectively [23, 55]. Further, in a study comparing several popular body dissatisfaction inventories, Davis, Lilly, Proctor, and Best [56] found that the BIQ correlated highly with other body dissatisfaction measures that are gender specific and, when used as a criterion variable in regression models, this scale accounted for the highest level of variability for males and females. The internal reliability of this scale was α = .80 for females and α = .82 for males in the current study.

#### Personality

Data sets from two samples were combined for the current purposes. The studies used one of two measures of personality; however, both measures reflect the FFM of personality and result in congruent factor scores. In the first study, the Neuroticism, Extraversion and Openness Personality Inventory-Revised (NEO-PI-R) [42, 43] was used. This measure is a 240-item self-report questionnaire that measures five factors of personality: Neuroticism; Extraversion; Openness to experience; Agreeableness; and, Conscientiousness. Each item is scored on a 5-point Likert scale ranging from 1 (*strongly disagree*) to 5 (*strongly agree*). This personality inventory is a reliable and valid self-report instrument used for assessing the personality domains of the Five-Factor Model in males and females [43]. In the second study, the NEO Five-Factor Inventory-3 (NEO-FFI-3) was administered. This measure is a 60-item self-report questionnaire that provides FFM Factor Scores [57]. The 60 FFI items are a sample of the 240 NEO-PI-R items but the instrument provides only summary factor scores rather than more specific facet level scores. There is a high correlation between the factor scores on these scales (*r >* .75) [43]. Each item is scored on a 5-point Likert scale ranging from 0 (*strongly agree*) to 4 (*strongly disagree*). The Adult version of this personality inventory is a reliable and valid self-report instrument for males and females [57]. For females in the current study, the internal reliabilities of the subscales were: α = .82 (Neuroticism); α = .80 (Extraversion); α = .64 (Openness to experiences); α = .78 (Agreeableness); and, α = .81 (Conscientiousness). The lower reliability of the Openness subscale is somewhat concerning but this factor was not a variable of interest in the regression analyses (this scale has consistently lower internal reliability than other factors). For males in the current study, the internal reliabilities of the subscales were: α = .87 (Neuroticism); α = .82 (Extraversion); α = .74 (Openness to experiences); α = .78 (Agreeableness); and, α = .84 (Conscientiousness).

## Results

### Sample characteristics

After screening the data set and removing univariate and multivariate outliers, the sample consisted of 323 university students (73.7% female), and the age of participants ranged from 17 to 47 years (*M* = 20.52, *SD* = 4.22). Among female participants, self-reported height ranged from 58 in. to 74 in. (*M* = 65.06, *SD* = 2.86) and self-reported weight ranged from 81 lb to 350 lb (*M* = 144.66, *SD* = 33.01), resulting in an average self-reported BMI of 24.02 kg/m^2^ (*SD* = 4.97). The self-reported height of male participants ranged from 59 in. to 80 in. (*M* = 71.14, *SD* = 3.31) and self-reported weight ranged from 110 lb to 320 lb (*M* = 177.79, *SD* = 33.85), resulting in an average self-reported BMI of 24.66 kg/m^2^ (*SD* = 4.21). The difference between the self-reported BMIs of women and men was not statistically significant. In addition, the correlation between age and BMI was not statistically significant, *r*(321) = .11, *p* = .07. Although previous researchers have reported that the relation between BMI and body dissatisfaction was nonlinear for male participants, in the current study a linear model best described the associations between BMI and dissatisfaction for both genders.

As expected, women reported greater levels of body dissatisfaction and disordered eating than men, with women scoring higher on the EAT-26, *t*(319) = 2.95, *p* = .003, *d* = 0.38, and BIQ, *t*(321) = 3.45, *p* = .001, *d* = 0.44. Gender differences were further explored by examining differences in personality traits. With regard to personality traits, women reported higher levels of both Neuroticism, *t*(321) = 5.34, *p* < .001, *d* = 0.68, and Agreeableness, *t*(321) = 3.86, *p* < .001, *d* = 0.49. See Table 1 for means and standard deviations for all measures.

**Table 1:** Means and standard deviations for all measures

	Females		Males	
	*M*	*SD*	*M*	*SD*
BMI	24.02	4.97	24.66	4.21
EAT-Total	14.87	12.05	10.69	8.13
BIQ	2.19	1.44	1.57	1.37
Neuroticism	2.25	0.56	1.87	0.58
Extraversion	2.36	0.54	2.45	0.52
Openness	2.48	0.47	2.47	0.45
Agreeableness	2.58	0.48	2.35	0.45
Conscientiousness	2.37	0.49	2.36	0.46

### Correlational relationships

Correlational relationships were examined first to determine which personality factors were most strongly related to body dissatisfaction and disordered eating in women and men. As suggested by previous research, among women, there was a statistically significant positive correlation between total EAT-26 scores and Neuroticism scores as well as statistically significant negative correlations between total EAT-26 scores and Agreeableness and Conscientiousness scores. Interestingly and contrary to consistent findings with women, levels of Neuroticism were not significantly related to total EAT-26 scores in men. In fact, none of the personality factors were significantly correlated with total EAT-26 scores in men (see Table 2).

**Table 2:** Correlations between BMI, NEO factor scores, EAT scores, BIQ scores

	EAT females	EAT males	BIQ females	BIQ males
BMI	.03	.01	.26**	.37**
Neuroticism	.20**	.21	.41**	.41**
Extraversion	.07	−.17	−.12	−.22*
Openness	−.12	−.14	−.09	−.21
Agreeableness	−.18**	−.15	−.14*	−.17
Conscientiousness	−.18**	.01	−.13*	−.31**
EAT			.42**	.19

*Note*. *p < 0.05, ** *p* < 0.01

Further, among women, there was a statistically significant positive correlation between BIQ and Neuroticism scores as well as statistically significant negative correlations between BIQ and Agreeableness and Conscientiousness scores. In men, there was a statistically significant positive correlation between BIQ scores and Neuroticism scores as well as negative correlations between BIQ scores and Extraversion and Conscientiousness scores. Interestingly, for women, BIQ scores were significantly positively correlated with EAT-26 scores but among men, BIQ scores were not significantly correlated with total EAT-26 scores (see Table 2).

### Prediction of body dissatisfaction

In order to determine if the sample size was adequate, we conducted a power analysis (using G-Power). Assuming a small effect size, power analysis results indicated that 84 participants would be necessary to achieve a power of .80, suggesting that analyses could be conducted separately for males and females. Prior to analysis, the dataset was screened for model assumptions and no violations were detected. Given that a nonlinear relation between BMI and body dissatisfaction has been reported, we examined linearity statistics as well as scatterplots between predictor and criterion variables with loess curves fitted for males and females. Overall, in assessing both BIQ and EAT-26 scores, any departures from linearity were quite small. Further, linear transformations did not improve the predictability of the models and, as such, for ease of interpretation and comparison, we used hierarchical linear regressions to compare gender differences in dissatisfaction and eating behaviours.

A series of four hierarchical multiple regression analyses were performed to assess the role of personality in the prediction of body dissatisfaction and disordered eating in women and men, separately. For both men and women, BIQ scores were entered as the criterion variable in the first analysis. BMI was entered on the first step to control for reported current body size. The five NEO factor scores were entered on the second step. Although many researchers choose to include gender as a Block 1 variable or as a moderating variable, we chose not to because we wanted to examine unique processes that are at work in male and female dissatisfaction. If added as a Block 1 variable, the overall model would be statistically significant, with gender adding statistically significant predictability to the model. As such, the effects of gender would be controlled in subsequent blocks. Although this is a valid way of considering the data, running separate analyses allows us to discern specific differences in the predictors of dissatisfaction in males and females.

Among female participants, the overall model was statistically significant, *R* ^*2*^ = .23, *F*(6, 226) = 11.53, *p* < .001. On the first step, BMI emerged as a statistically significant predictor of body dissatisfaction, *R* ^*2*^ = .07, *F*(1, 231) = 17.31, *p* < .001, and after controlling for BMI, Neuroticism scores accounted for the most unique variance, *β* = .40, *t*(228) = 6.48, *p* < .001 (see Table 3). For males, the overall model was also statistically significant, *R* ^*2*^ = .42 *F*(6, 77) = 9.18, *p* = .001. On the first step, BMI emerged as a statistically significant predictor of body dissatisfaction, *R* ^*2*^ = .13, *F*(1, 82) = 13.15, *p* < .001, and after controlling for BMI, Neuroticism scores accounted for the most unique variance, *β* = .35, *t*(82) = 3.53, *p* = .001, followed by Conscientiousness scores, *β* = −.19, *t*(82) = −2.15, *p* = .035 (see Table 3).

**Table 3:** Summary of regression analyses for the prediction of body dissatisfaction in females and males

	*B*	SE(*B*)	*β*
Female participants			
*Step 1*			
BMI	.08	.02	.26**
*Step 2*			
Neuroticism	.10	.16	.40**
Extraversion	.12	.17	.05
Openness to experience	−.04	.19	−.01
Agreeableness	−.13	.19	−.04
Conscientiousness	−.19	.18	−.07
Male participants			
*Step 1*			
BMI	.12	.03	.37**
*Step 2*			
Neuroticism	.83	.24	.35**
Extraversion	−.15	.26	−.06
Openness to Experience	−.40	.28	−.13
Agreeableness	−.41	.28	−.14
Conscientiousness	−.58	.27	−.19*

*Note*. * *p* < .05, ** *p* < .01

To examine specific differences in eating attitudes, similar analyses were conducted. Total EAT scores were entered as the criterion variable in the second analysis. BMI was entered on the first step to control for reported current body size. NEO factor scores were entered on the second step. For female participants, the overall model was significant, *R* ^*2*^ = .11, *F*(6, 225) = 4.56, *p* = .001. Although BMI was not a statistically significant predictor of disordered eating in this model, Neuroticism scores accounted for the most unique variance, *β* = .22, *t*(225) = 2.29, *p* = .001, followed by Extraversion, *β* = .17, *t*(225) = 2.52, *p* = .012 and Conscientiousness scores, *β* = −.15, *t*(225) = −2.31, *p* = .024 (see Table 4). For male participants, the overall model was not significant, indicating that BMI and NEO factor scores did not predict EAT scores in males (see Table 4).

**Table 4:** Summary of regression analyses for the prediction of disordered eating in females and males

	*B*	SE(*B*)	*β*
Female participants			
*Step 1*			
BMI	.07	.16	.027
*Step 2*			
Neuroticism	4.79	1.46	.22**
Extraversion	3.86	1.52	.17**
Openness to experience	−2.34	1.71	−.09
Agreeableness	−2.79	1.70	−.11
Conscientiousness	−3.73	1.62	−.15**
Male participants			
*Step 1*			
BMI	.03	.22	.01
*Step 2*			
Neuroticism	2.80	1.76	.20
Extraversion	−.942	1.97	−.06
Openness to Experience	−1.43	2.1	−.08
Agreeableness	−2.06	2.06	−.12
Conscientiousness	1.26	2.02	.07

*Note*. * *p* < .05, ** *p* < .01

## Discussion

Much research has explored personality correlates of body dissatisfaction and disordered eating in women, and a high level of neuroticism is most consistently associated with these variables [44–47, 50–52, 58–60]. It was hypothesized that personality factor scores would predict body dissatisfaction for women, and this was supported in the current study. Specifically, higher levels of neuroticism predicted higher levels of body image dissatisfaction, which replicates previous findings in women [44, 47, 58, 60]. The current research extends this relation to men, as higher levels of neuroticism and lower levels of conscientiousness predicted higher levels of body image dissatisfaction in male participants. Generally, women report higher levels of neuroticism than men, and this was supported in the current study; therefore, neuroticism could act as a mediating factor in the relationship between gender and body dissatisfaction, such that women have higher levels of neuroticism, and thereby exhibit higher levels of body image dissatisfaction [61].

It was also hypothesized that personality factor scores would predict levels of disordered eating in women, and this was supported in the current study. Specifically, higher levels of neuroticism and lower levels of conscientiousness predicted higher levels of disordered eating, which is in line with previous findings with women [44, 47, 58, 60]. This relationship was not extended to men in the current study, as personality factor scores did not predict levels of disordered eating in men. These results suggest that women with disordered eating attitudes and behaviours may be more prototypical with regard to previous conceptualizations of disordered eating, compared to men. In line with conclusions made by Farstad and colleagues [15], we suggest that the consideration of personality traits in the treatment of body image and eating concerns may contribute to a better understanding of their etiology and maintenance as well as inform the development and selection of interventions that are sensitized to individual variation. Indeed, previous research has demonstrated that questionnaires assessing the Big Five traits (e.g., NEO-PI-R) have demonstrated predictive utility for treatment results and symptom variation among individuals with an Eating Disorder [15, 62].

Aside from observed dissimilarities relating to personality and disordered eating variables, gender differences were evident with regard to contributions of BMI in the prediction of body image dissatisfaction. Specifically, for females, BMI did not predict a large amount variance in body dissatisfaction (7%) compared to males (14%). These results suggest that a men’s weight-to-height ratio is a stronger predictor of body dissatisfaction than in women. Generally, males who were bigger in size (i.e., larger BMI) were more dissatisfied with their appearance. On the other hand, although statistically significant, BMI was not as substantial a predictor of body dissatisfaction in women. This could be because many women, regardless of shape or size, are dissatisfied with their bodies [63].

Men may experience body dissatisfaction and/or disordered eating for different or more variable reasons than females. It is interesting to note that among male participants, the relation between body dissatisfaction and disordered eating was not statistically significant. These results warrant further examination as it appears that the processes underlying the etiology of specific eating disorders may be different for males and females. Although body dissatisfaction can serve as a warning sign for problems with eating among females, it same may not be true for males. These results highlight the importance of well-validated assessment tools that take gender specific body image and disordered eating concerns into account. For instance, although there are several male specific body image measures including: Male Body Attitudes Scale [64]; Drive for Muscularity Scale [65]; Muscle Appearance Satisfaction Scale [66]; Male Body Checking Questionnaire [67]; and, the Male Body Dissatisfaction Scale [68], these measures are designed explicitly for males, making it difficult to explore gender differences. To illustrate, in a comparison of five scales that assess body image dissatisfaction, the Body Image Ideals Questionnaire was found to account for the most variability in female body image dissatisfaction, whereas the Male Body Dissatisfaction scale accounted for the most variability in male body dissatisfaction [56]. Further, measures of disordered eating do not consist of questions relating to excessive exercise, or alternative weight control strategies that could be utilized by males. Findings such as these emphasize the need for scales that accurately measure dissatisfaction and disordered eating behaviours in both males and females.

Results of the current study should be considered in light of several limitations. First, the current sample was nonclinical and consisted mostly of young Caucasian university students. Therefore, these data should be extrapolated to other groups of individuals with caution. Second, although a power analysis indicated adequate power for all analyses, the current sample contained a low number of male participants in comparison to female participants. Although this gender distribution is common in psychological research, the power of the results would be strengthened if a higher proportion of male participants were included. Third, our predictor variables were selected because they are salient factors associated with body dissatisfaction and disordered eating. In spite of the fact that each of our regression models led to statistically significant *R* ^2^ values, effect sizes for males and females were small and there was a large proportion of unaccounted variability in these outcome variables.

Aside from the Big Five personality traits, other personality factors have been associated with eating disorders and associated concerns (e.g., body dissatisfaction) in males and females. Examples include perfectionism, impulsiveness, and approach and avoidance motivation [15]. Future research should continue to explore relations between disordered eating, body image, and diverse personality constructs. Although the present study focused on the FFM of personality, other models of personality could be explored by administering alternative measures of personality such as the Eysenck Personality Questionnaire [69] or the Temperament Character Inventory [70].

Despite these above limitations, the current study was able to compare and contrast factors associated with body image and disordered eating in women and men in the same study. Upon examination of our results, it is evident that important gender differences exist in the experience of disordered eating and body dissatisfaction in a nonclinical university population. More studies investigating factors related to body image and eating concerns in males and females are needed to corroborate these findings.

## Conclusions

Consistent associations between personality factors, disordered eating, and body dissatisfaction have been drawn largely from female samples and the current research highlights the importance of considering male participants. A key finding is the lack of relationship between disordered eating and personality in men, which is a well-established association in women. Although previous studies have estimated that males account for somewhere between 5 and 15% of patients with eating disorders [10, 28], in the current study men accounted for over 18% of participants reporting symptoms of disordered eating. If the number of men with disordered eating is increasing, it is imperative to investigate factors which predict this symptomology in men. Furthermore, among men, one of the best predictors of body dissatisfaction is current body size, with larger bodies predicting more body dissatisfaction. Therefore, weight management strategies, for example, may be more appropriate for targeting these issues in men than in women. Overall, these gender differences could have potential clinical relevance, which warrant further investigation.

## References

[1] Catikkas F (2011). Physical correlates of college students’ body image satisfaction levels. J Soc Behav Person.

[2] Frederick DA, Forbes GB, Grigorian KE, Jarcho JM (2007). The UCLA body project I: gender and ethnic differences in self-objectification and body satisfaction among 2,206 undergraduates. Sex Roles.

[3] Hoyt WD, Kogan LR (2002). Satisfaction with body image and peer relationships for males and females in a college environment. Sex Roles.

[4] Beebe DW (1995). The attention to body shape scale: a new measure of body focus. J Pers Assess.

[5] Lokken K, Ferraro FR, Kirchner T, Bowling M (2003). Gender differences in body size dissatisfaction among individuals with low, medium, or high levels of body focus. J Gen Psychol.

[6] McDonald K, Thompson JK (1992). Eating disturbance, body image dissatisfaction, and reasons for exercising: gender differences and correlational findings. Int J Eat Disord..

[7] Mellor D, Fuller-Tyszkiewicz M, McCabe M, Ricciardelli L (2010). Body image and self-esteem across age and gender: a short-term longitudinal study. Sex Roles.

[8] Stanford JN, McCabe MP (2002). Body image ideal among males and females: sociocultural influences and focus on different body parts. J Health Psychol.

[9] Cash TF, Deagle EA (1997). The nature and extent of body-image disturbances in anorexia nervosa and bulimia nervosa: a meta-analysis. Int J Eat Disord..

[10] Fernandez-Arando F, Aitken A, Badia A, Gimenez L, Solano R, Collier D (2004). Personality and psychopathological traits of males with an eating disorder. Eur Eat Disord Rev.

[11] Lewinsohn PM, Seeley JR, Moerk KC, Striegel-Moore R (2002). Gender differences in eating disorder symptoms in young adults. Int J Eat Disord..

[12] Stice E, Shaw HE (2002). Role of body dissatisfaction in the onset and maintenance of eating pathology: a synthesis of research findings. J Psychosom Res.

[13] Polivy J, Herman P (2002). Causes of eating disorders. Annu Rev Psychol.

[14] Cassin SE, von Ranson KM (2005). Personality and eating disorders: a decade in review. Clin Psychol Rev.

[15] Farstad SM, McGeown LM, Eating v RKM (2016). Disorders and personality, 2004-2016: a systematic review and meta-analysis. Clin Psychol Rev.

[16] Fredrickson BL, Roberts TA (1997). Objectification theory: toward understanding women’s lived experiences and mental health risks. Psychol Women Q.

[17] López-Guimera G, Levine MP, Sánchez-Carracedo D, Fauquet J (2010). Influence of mass media on body image and eating disordered attitudes and behaviours in females: a review of effects and processes. Media Psychol.

[18] Thompson JK, Heinberg LJ, Altabe M, Tantleff-Dunn S (1999). Exacting beauty: theory, assessment, and treatment of body image disturbance.

[19] Brechan I, Kvalem IL (2015). Relationship between body dissatisfaction and disordered eating: mediating role of self-esteem and depression. Eat Behav.

[20] Fallon AE, Rozin P (1998). Sex differences in perceptions of desirable body shape. J Abnorm Psychol.

[21] Lamb CS, Jackson LA, Cassiday PB, Priest DJ (1993). Body figure preferences of men and women: a comparison of two generations. Sex Roles.

[22] Grieve FG, Newton CC, Kelley L, Miller RC, Kerr NA (2005). The preferred male body shapes of college men and women. Individ Differ Res.

[23] Muth JL, Cash TF (1997). Body image attitudes: what difference does gender make?. J Appl Soc Psychol.

[24] Hesse-Biber S, Clayton-Matthews A, Downey JA (1987). The differential importance of weight and body-image among women and men. Genet Soc Gen Psychol Monogr.

[25] Kashubeck-West S, Mintz LB, Weigold I (2005). Separating the effects of gender and weight-loss desire on body satisfaction and disordered eating behavior. Sex Roles.

[26] Ousley L, Cordero ED, White S (2008). Eating disorders and body image of undergraduate men. J Am Coll Heal.

[27] American Psychiatric Association (2013). Diagnostic and statistical manual of mental disorders.

[28] Stoving RK, Andries A, Brixen K, Bilenberg N, Horder K (2011). Gender differences in outcome of eating disorders: a retrospective cohort study. Psychiatry Res.

[29] Lindvall DC, Wisting L (2016). Transitioning from DSM-IV to DSM-5: a systematic review of eating disorder prevalence assessment. Int J Eat Disord..

[30] Mancilla-Diaz JM, Franco-Paredes K, Vazquez-Arevalo R, Lopez-Aguilar X, Alvarez-Rayon GL, Tellez-Giron MTA (2007). Two-stage epidemiologic study on prevalence of eating disorders in female university students in Mexico. Eur Eat Disord Rev.

[31] Pelaez Fernandez MA, Labrador FJ, Raich RM (2007). Prevalence of eating disorders among adolescent and young adult scholastic population in the region of Madrid (Spain). J Psychosom Res.

[32] Vardar E, Erzengin M (2011). The prevalence of eating disorders (EDs) and comorbid psychiatric disorders in adolescents: a two-stage community-based study. Turk Psikiyatri Derg.

[33] Anderson C, Bulik CM (2004). Gender differences in compensatory behaviors, weight and shape salience, and drive for thinness. Eat Behav.

[34] Striegel-Moore RH, Rosselli F, Perrin N, DeBar L, Wilson GT, May A, Kraemer HC (2009). Gender difference in the prevalence of eating disorder symptoms. Int J Eat Disord..

[35] Jones WR, Morgan JF (2010). Eating disorders in men: a review of the literature. J Public Ment Health.

[36] Ricciardelli LA, Wade T (2017). Eating disorders in boys and men. Encyclopedia of feeding and eating disorders.

[37] Weltzin TE, Weisensel N, Franczyk D, Burnett K, Klitz C, Bean P (2005). Eating disorders in men: Update. J Mens Health Gend.

[38] Farquhar JC, Wasylkiw L (2007). Media images of men: trends and consequences of body conceptualization. Psychol Men Masc.

[39] Thompson JK, Cafri G, Thompson JK, Cafri G (2007). The muscular ideal: an introduction. The muscular ideal.

[40] Digman JM (1990). Personality structure: emergence of the five-factor model. Annu Rev Psychol.

[41] Norman WX (1963). Toward an adequate taxonomy of personality attributes: replicated factor structure in peer nomination personality ratings. J Abnorm Soc Psychol.

[42] Costa PT, McCrae RR (1985). The NEO personality inventory manual.

[43] Costa PT, McCrae RR (1992). Normal personality assessment in clinical practice: the NEO personality inventory. Psychol Assess.

[44] Dalley SE, Buunk AP, Umit T (2009). Female body dissatisfaction after exposure to overweight and thin media images: the role of body mass index and neuroticism. Pers Individ Dif.

[45] MacLaren VV, Best LA (2009). Female students’ disordered eating and the big five personality facets. Eat Behav.

[46] Miller JL, Schmidt LA, Louis A, Vaillancourt T, McDougall P, Laliberte M (2006). Neuroticism and introversion: a risky combination for disordered eating among a non-clinical sample of undergraduate students. Eat Behav.

[47] Roberts A, Good E (2010). Media images and female body dissatisfaction: the moderating effects of the five-factor traits. Eat Behav.

[48] Swami V, Buchanan T, Furnham A, Tovee MJ (2008). Five-factor personality correlates of perceptions of women’s body sizes. Person Individ Dif..

[49] Swami V, Hurnham A, Chamorro-Premuzic T, Akbar K, Gordon N, Harris T (2010). More than just skin deep? Personality information influences men’s ratings of the attractiveness of women’s body sizes. J Soc Psychol.

[50] Claridge G, Davis C (2001). What’s the use of neuroticism?. Person Individ Dif.

[51] Tylka TL (2004). The relation between body dissatisfaction and eating disorder symptomatology: an analysis of moderating variables. J Couns Psychol.

[52] Swami V, Taylor R, Carvalho C (2011). Body dissatisfaction assessed by the photographic figure rating scale is associated with sociocultural, personality, and media influences. Scand J Psychol.

[53] Garner DM, Olmsted MP, Bohr Y, Garfinkel PE (1982). The eating attitudes test: psychometric features and clinical correlates. Psychol Med.

[54] Garfinkel PE, Newman A (2001). The eating attitudes test: twenty-five years later. Eat Weight Disord.

[55] Cash TF. Body ideals questionnaire. 2000. http://www.body-images.com/.

[56] Davis L, Proctor C, Lilly S, Best LA, Pracana C (2016). Body dissatisfaction: effects of gender, exercise, personality, and disordered eating. Proceedings of the international psychological applications conference and trends.

[57] McCrae RR, Costa PT (2010). NEO inventories professional manual for the NEO personality Inventory-3, NEO five-factor Inventory-3, and NEO personality inventory revised.

[58] Bergstrom RL, Neighbors C, Malheim J (2009). Media comparisons and threats to body image: seeking evidence of self-affirmation. J Soc Clin Psychol.

[59] Davis C, Dionne M, Lazarus L (1996). Gender-role orientation and body image in women and men: the moderating influence of neuroticism. Sex Roles.

[60] Groesz LM, Levine MP, Murnen SK (2002). The effect of experimental presentation of thin images on body satisfaction: a meta-analytic review. Int J Eat Disord.

[61] Guo S (1995). Sex differences in personality: a meta-analysis based on “big five” factors.

[62] Deumens RA, Noorthoorn EO, Verbraak MJ (2012). Predictors for treatment outcome of binge eating with obesity: a naturalistic study. Eat Disord.

[63] Tiggemann M, Cash TF, Smolak L (2011). Sociocultural perspectives on human appearance and body image. Body image: a handbook of theory, research, and clinical practice.

[64] Tylka TL, Bergeron D, Schwartz JP (2005). Development and psychometric evaluation of the male body attitudes scale (MBAS). Body Image.

[65] McCreary DR, Sasse DK (2000). An exploration of the drive for muscularity in adolescent boys and girls. J Am Coll Heal.

[66] Mayville SB, Williamson DA, White MA, Netemeyer RG, Drab DL (2002). Development of the muscle appearance satisfaction scale: a self-report measure for the assessment of muscle dysmorphia symptoms. Assessment.

[67] Hildebrandt T, Walker DC, Alfano L, Delinsky S, Bannon K (2010). Development and validation of a male specific body checking questionnaire. Int J Eat Disord..

[68] Ochner N, Gray JA, Brickner K (2009). The development and initial validation of a new measure of male body dissatisfaction. Eat Behav.

[69] Eysenck JJ, Eysenck SBG (1991). Eysenck personality questionnaire-revised (EPQ-R).

[70] Cloninger RC (1994). The temperament and character inventory (TCI): a guide to its development and use.

